# The Artificial Pneumothorax Operation

**Published:** 1914-02-21

**Authors:** H. de Carle Woodcock, J. A. M. Clark


*To the Editor of* The Hospital.

### The Artificial Pneumothorax Operation.

*To the Editor of* The Hospital

Sir,—Referring to your notes on our work on artificial pneumothorax (The Hospital, February 7), we should like to draw attention to some further points of practical importance in this connection.

The principle on which the practice of the production of artificial pneumothorax is based is an old one: the application of rest to an organ that is diseased. But the operator must not think that the extensiveness of the disease is a measure of the suitability of the case for operation. There are some conditions in which the operation is an exceedingly dangerous one. We have certainly seen an acute bronchitis come on in an emphysematous patient with phthisis who was subject to occasional attacks of catarrhal bronchitis.

Now we have frequently induced an artificial pneumothorax when faced with the knowledge that our patient had a very large, or even more than one very large, cavity. If one lung is anything like healthy the presence of large cavities in the other lung does not deter us from doing the operation, but we repeat that we never operate where there is a constant dyspnœa present. Cardiac irregularity, or even disease, does not of itself prevent us from operating; marked cyanosis does. Unilateral disease invites the operation, but bilateral disease does not necessarily bar its performance. Laryngeal tuberculosis is generally greatly relieved by the operation of pneumothorax. In some cases it is made worse—especially directly after the operation. If a pneumothorax is doing good the signs of cavitation should be lessening, and the amount of sputum considerably diminished or entirely abolished. In one patient, where recovery was quite hopeless, the sputum was very much lessened and the comfort of the patient increased.

The instrument which we use is the simplest that we have seen, and includes an arrangement for making nitrogen gas. The nitrogen is produced by passing the atmospheric air through a solution of caustic soda and pyrogallate of potash. The method of introducing the nitrogen gas into the pleura is to pass a hollow needle through the chest-wall, arranging that the needle is connected with an indiarubber tube, through which the gas can be driven from the gas-bottle. In effect, you make the gas-bottle an annexe of the pleural cavity, and then displace the gas from the bottle into the pleural cavity by filling the former with water. A three-way cock is attached to the indiarubber tube, which opens to the manometer. When the needle has penetrated the pleural cavity, the manometer registers a negative pressure, for the pressure in the pleural cavity is, of course, negative. The patient may die from gas embolism; he may die from shock. The oscillation of the fluid in the manometer guards against embolism, for there is no oscillation when the needle has penetrated a vein. The patient may, as we have said, die from shock, from pleuro-cardiac reflex due to too great a stimulus to the nervous mechanism of the pleura, by which the action of the heart may be suddenly and profoundly embarrassed, even to the extent of death. The use of local anæsthetics (*e.g.*, novocain and adrenalin) and the introduction of urea-hydrochloride of quinine into the deeper tissues prevent pleuro-cardiac shock. During an operation it is essential that one assistant should watch carefully for indications of catastrophe. If the pulse becomes irregular or quick, the breathing embarrassed or slow or gaping, if the face changes colour, especially if it goes grey, the operation must be immediately stopped, stimulants given, and gas allowed to escape through the pneumothorax needle.

We have had no deaths, but it is evident that once or twice we have been very near disaster. On the other hand, very many patients are now living who otherwise would not be living; indeed, one of our worst cases was anxious to be able on Christmas Day to waltz, and begged to be allowed to do so. It is evident from our experience that the operation is very valuable, and that it may be done in many advanced cases. If the contra-indications that we have just mentioned are not present, it should most certainly be done, but it is more successful in unilateral disease; small cavities may be obliterated by it, and large cavities may in many cases be greatly lessened in size. When the cavitation signs have been abolished, or greatly and favourably modified, the treatment should be suspended. The healthy lung to a great extent expands again, and the process of fibrotic change around and in the diseased area or areas prevents re-expansion of cavities and consequent revival of clinical symptoms.—We are faithfully yours,

H. de Carle Woodcock, M.D., M.R.C.P., D.P.H.
J. A. M. Clark, M.D., D.P.H.

